# Antibody responses to SARS-CoV-2 variants LP.8.1, LF.7.1, NB.1.8.1, XFG, and BA.3.2 following KP.2 monovalent mRNA vaccination

**DOI:** 10.1128/mbio.02901-25

**Published:** 2025-11-25

**Authors:** Anass Abbad, Brian Lerman, Jordan Ehrenhaus, Brian Monahan, Gagandeep Singh, Adria Wilson, Stefan Slamanig, Ashley Aracena, Neko Lyttle, Jessica R. Nardulli, Keith Farrugia, Zain Khalil, Ana Silvia Gonzalez-Reiche, Mia Emilia Sordillo, Weina Sun, Harm van Bakel, Viviana Simon, Florian Krammer

**Affiliations:** 1 Department of Microbiology, Icahn School of Medicine at Mount Sinai 5925, New York, New York, USA; 2 Center for Vaccine Research and Pandemic Preparedness (C-VaRPP), Icahn School of Medicine at Mount Sinai 5925, New York, New York, USA; 3 Department of Genetics and Genomic Sciences, Icahn School of Medicine at Mount Sinai 5925, New York, New York, USA; 4 Department of Pathology, Molecular and Cell Based Medicine, Icahn School of Medicine at Mount Sinai 5925, New York, New York, USA; 5 Department of Artificial Intelligence and Human Health, Icahn School of Medicine at Mount Sinai, New York, New York, USA; 6 Icahn Genomics Institute, Icahn School of Medicine at Mount Sinai, New York, New York, USA; 7 The Global Health and Emerging Pathogens Institute, Icahn School of Medicine at Mount Sinai 5925, New York, New York, USA; 8 Division of Infectious Diseases, Department of Medicine, Icahn School of Medicine at Mount Sinai 5925, New York, New York, USA; 9 Ludwig Boltzmann Institute for Science Communication and Pandemic Preparedness at the Medical University of Vienna, Medical University of Vienna 27271, Vienna, Austria; 10 Ignaz Semmelweis Institute, Interuniversity Institute for Infection Research, Medical University of Vienna, Vienna, Austria; Tsinghua University, Beijing, China

**Keywords:** SARS-CoV-2, COVID-19, Omicron, mRNA vaccine, antigenic cartography

## Abstract

The evolution of severe acute respiratory syndrome coronavirus 2 (SARS-CoV-2) has resulted in antigenically distinct variants that challenge vaccine-induced immunity. The KP.2 monovalent mRNA vaccine was deployed in 2024 to address immune escape by emerging SARS-CoV-2 subvariants. We assessed neutralizing antibody responses in 56 adults with varied exposure histories following KP.2 vaccination against emerging variants including LP.8.1, LF.7.1, NB.1.8.1, XFG, and BA.3.2. While KP.2 vaccination enhanced neutralization against homologous variants, substantial reductions in neutralizing activity were observed against emerging Omicron variants across all exposure groups. Exposure history showed some influence on neutralization breadth, with self-reported vaccination-only participants exhibiting better cross-neutralization compared to individuals with hybrid immunity. Antigenic cartography revealed substantial antigenic distances between KP.2 and emerging variants, highlighting significant immune escape potential that threatens vaccine protection.

**IMPORTANCE:**

Severe acute respiratory syndrome coronavirus 2 (SARS-CoV-2) continues to evolve, producing variants that escape vaccine-induced immunity. The current work shows that KP.2 monovalent vaccination provides limited protection against antigenically distant Omicron variants (LP.8.1, LF.7.1, NB.1.8.1, XFG and BA.3.2). These findings highlight the ongoing challenge of maintaining vaccine effectiveness against evolving SARS-CoV-2 variants and argue for continuous updating of vaccines.

## OBSERVATION

Severe acute respiratory syndrome coronavirus 2 (SARS-CoV-2) continues to evolve in immunologically experienced populations, with emerging variants demonstrating enhanced immune escape. The KP.2 monovalent mRNA vaccine was deployed in 2024 (1). A recombinant protein-based vaccine with the spike of a very similar variant, JN.1, will likely be used in the 2025/2026 season as well, even though the mRNA vaccines have been updated to LP.8.1. Understanding neutralizing antibody responses against emerging variants is crucial for informing vaccine strategy and pandemic preparedness. Here, we assessed neutralizing and binding antibody responses in 56 adult study participants with varied SARS-CoV-2 exposure histories following KP.2 monovalent vaccination.

**Fig 1:**
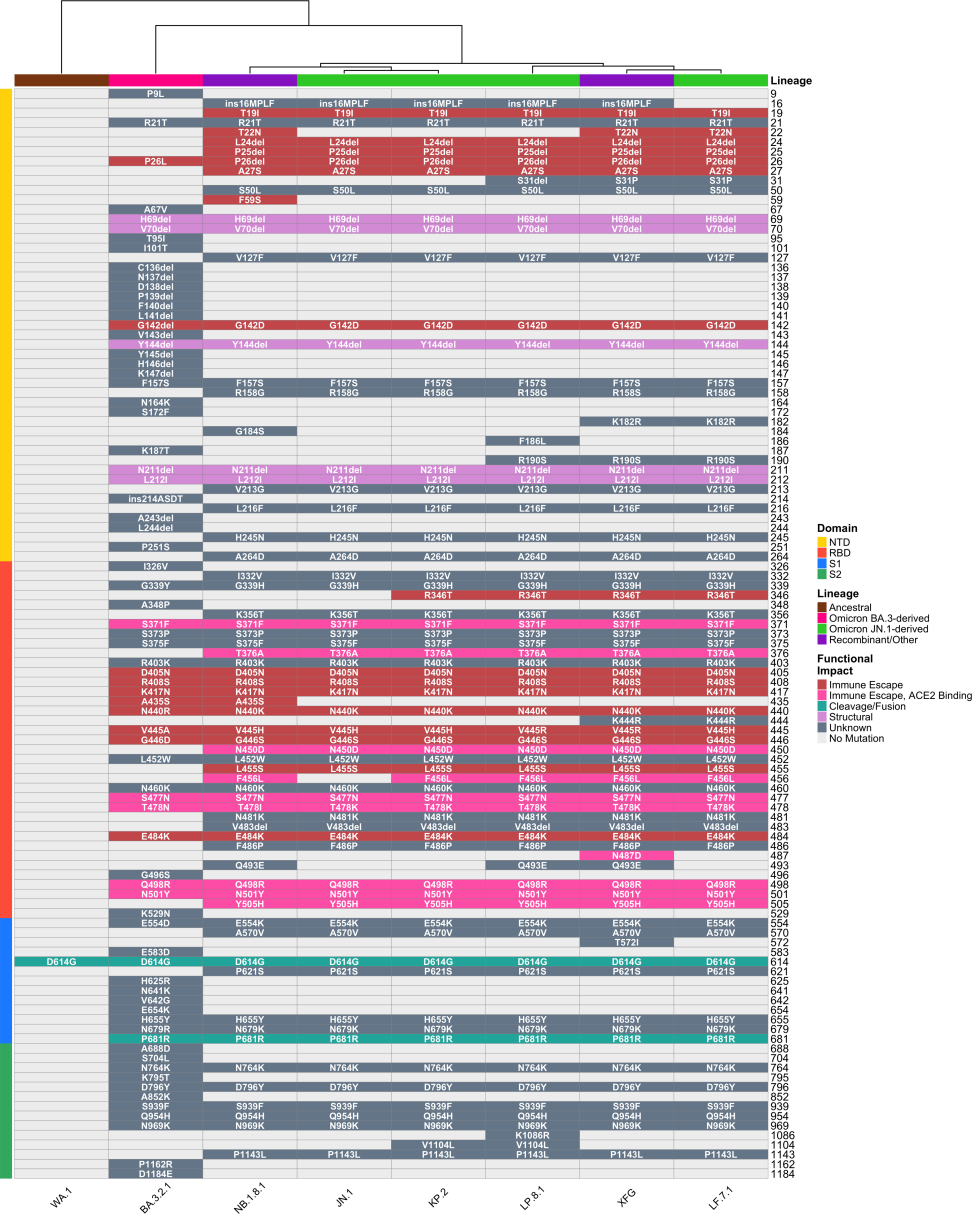
SARS-CoV-2 spike mutations across major variants. Heatmap displaying amino acid mutations in the SARS-CoV-2 spike protein across eight major variants (WA.1, JN.1, KP.2, LP.8.1, LF.7.1, NB.1.8.1, XFG, and BA.3.2) colored by predicted functional impact on receptor binding, antibody escape, and structural stability. Variants are organized by evolutionary lineage, with spike protein domains (N-terminal domain, receptor-binding domain, S1 and S2) indicated. Mutations shown correspond to the spike sequences of viral isolates used in neutralization assays. Dendrogram illustrate antigenic similarity relationships based on mutation patterns, with variants clustering by phylogenetic relationship. Colors reflect predicted functional consequences for each substitution.

We enrolled 56 healthy adults who received KP.2 monovalent mRNA vaccines and stratified them by exposure profile: vaccination-only (*n* = 16, self-reported, with no strong evidence of any past infection and with non-reactive ancestral SARS-CoV-2 anti-nucleoprotein [NP] binding antibodies), post-infection boosted (*n* = 11, booster vaccination soon after an infection), and complex hybrid immunity (*n* = 29, ≥2 infections plus ≥3 vaccine doses) (Table S1). Serum samples were collected an average of 29 days post-vaccination. We measured neutralizing antibodies against the ancestral WA.1 strain, vaccine-matched KP.2, JN.1, and emerging variants including LP.8.1, LF.7.1, NB.1.8.1, XFG, and BA.3.2 using live and pseudotyped virus microneutralization assays. The tested variants harbor distinct mutation patterns in key antigenic sites (Fig. 1). BA.3.2, a saltation variant with >50 mutations relative to BA.3, was originally identified in South Africa but has subsequently been detected globally (e.g., Germany, the Netherlands, California, and Australia), reflecting its capacity for transmission despite antigenic divergence (2, 3). FLiRT (named for the key F456L and R346T mutations) variants contain critical mutations including R346T, F456L, and Q498R that enhance both angiotensin converting enzyme 2 (ACE2) binding and antibody evasion.

**Fig 2:**
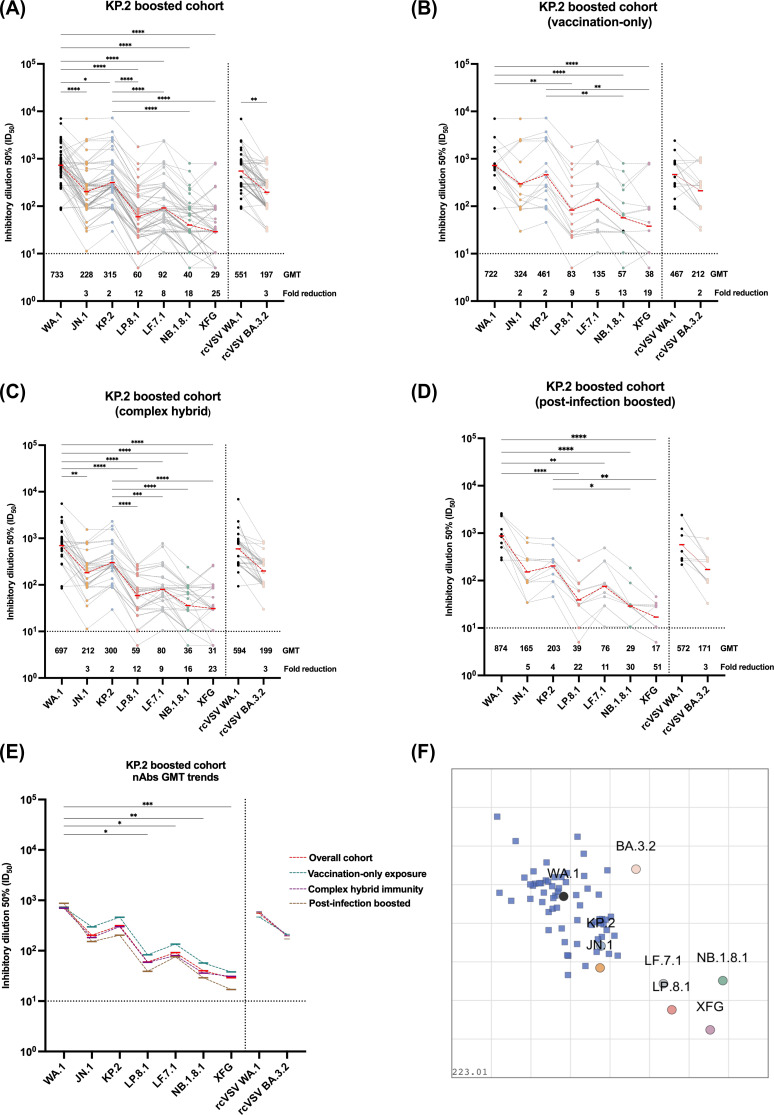
Neutralizing antibody responses and antigenic relationships following KP.2 vaccination. (**A**) Neutralizing antibody titers following KP.2 monovalent vaccination across exposure groups against the variant panel for the overall cohort, (**B**) vaccination-only group, (**C**) complex hybrid immunity group and (**D**) recent infection hybrid group. (**E**) Comparative GMT trends across all exposure groups overlaid on a single graph, showing neutralization patterns against each tested variant. Dashed lines connect GMT values for each group. Data are presented as aligned dot plots with individual participant responses connected by lines across the tested virus panel, allowing visualization of individual neutralization patterns. Statistical comparisons between variants within each exposure group are shown above brackets, with significance (ns : *P*-value >0.05, * : *P*-value > 0.05, **: *P*-value ≤ 0.01, ***: *P*-value ≤ 0.001 and **** : *P-*value ≤ 0.0001) determined by one-way ANOVA with Dunnett's multiple comparisons test. (**F**) Antigenic cartography reveals spatial relationships between SARS-CoV-2 variants. Two-dimensional antigenic map constructed from neutralizing antibody titers of the overall KP.2-boosted cohort against the tested variant panel. Each circle represents a virus variant, and each square a unique serum biospecimen, with distances proportional to antigenic differences based on neutralization data. Closely related variants cluster together, while antigenically distinct variants occupy distant positions. Grid lines represent 2-fold changes in neutralizing antibody titers, with each unit corresponding to a 2-fold difference. The map illustrates the antigenic landscape surrounding KP.2, highlighting the substantial antigenic drift of recent Omicron variants (LP.8.1, LF.7.1, NB.1.8.1, XFG) and the intermediate positioning of BA.3.2 relative to the vaccine and ancestral strains.

KP.2 vaccination enhanced neutralization against homologous KP.2 (GMT: 315) and closely related JN.1 (GMT: 203) variants (Fig. 2A through D). However, post-KP.2 vaccine sera showed substantially reduced neutralization against FLiRT variants across all exposure groups, with 12-fold reductions for LP.8.1, eightfold for LF.7.1, 18-fold for NB.1.8.1, and 25-fold for XFG compared to WA.1. Neutralizing titers against WA.1 (GMT: 733) significantly exceeded those against LP.8.1 (GMT: 60, *P* < 0.0001), LF.7.1 (GMT: 92, *P* < 0.0001), NB.1.8.1 (GMT: 40, *P* < 0.0001), and XFG (GMT: 29, *P* < 0.0001). BA.3.2 showed intermediate neutralization (GMT: 197), representing a 3-fold reduction compared to the ancestral strain.

Exposure history influenced neutralization breadth (Fig. 2E). Participants with self-reported vaccination-only immunity exhibited the most potent and broad responses, with highest titers against KP.2 (GMT: 461), JN.1 (GMT: 298), and better neutralization of FLiRT variants compared to other groups (Fig. 2B). Conversely, post-infection boosted participants exhibited lower cross-neutralization against LP.8.1 (GMT: 39), LF.7.1 (GMT: 76), NB.1.8.1 (GMT: 29), and XFG (GMT: 17) (Fig. 2D). Participants with complex hybrid immunity showed intermediate patterns across all variants (Fig. 2C). Importantly, it is unclear if these differences are big enough to be biologically meaningful, and they could also be an artifact of a small number of subjects tested. For these reasons, they should not be over-interpreted and require confirmation.

Antigenic cartography quantified these escape patterns (4, 5), revealing that emerging variants occupy distant positions in antigenic space relative to KP.2 (Fig. 2F). FLiRT variants clustered at antigenic distances exceeding three units from KP.2 (representing >8-fold neutralization reductions), while BA.3.2 occupied an intermediate but distinctly separate position. This spatial organization directly correlates with neutralization data and shows substantial immune escape potential that threatens protection.

Our findings reveal significant challenges posed by continued SARS-CoV-2 antigenic evolution. The substantial reduction in neutralizing activity against FLiRT variants, driven by mutations in critical antigenic sites, highlights the enhanced immune escape capabilities of these variants. The unexpected finding that vaccination-only participants showed better cross-neutralization compared to individuals with hybrid immunity challenges conventional assumptions about hybrid immunity advantages. However, this finding should not be over-interpreted as the differences were small and it is not clear if they represent biologically meaningful differences. Despite being NP antibody negative (Fig. S1) and having no self-reported infections, these individuals could of course have had asymptomatic/undetected infections. Furthermore, the finding could also be an artifact due to the small sample size.

Only an intermediate neutralization reduction was observed for BA.3.2, despite its extensive mutation profile. Our finding here is in contrast to another report (3) that shows more drastic reduction in neutralization. This difference may be explained by different assay settings. Specifically, we used an assay that assesses multicycle replication in the presence of serum, while other reports essentially only looked at initial entry inhibition. The results are in better agreement with a study from Germany (2), even though the variant comparisons are not exactly the same. Our results may explain why this variant has not achieved high transmission rates globally. This could reflect a balance between immune escape and viral fitness costs associated with extensive mutations.

These data highlight the need for adaptive vaccine approaches. While our study focused on humoral immunity, SARS-CoV-2-specific T-cell responses exhibit substantial cross-reactivity across Omicron variants, recognizing conserved epitopes less affected by spike mutations (6, 7). This preserved cellular immunity likely contributes to continued protection against severe COVID-19 despite reduced neutralizing antibody titers. Future strategies should consider targeting conserved epitopes or employing alternative delivery methods such as intranasal vaccination to enhance mucosal protection (8). Continuous antigenic surveillance and rapid vaccine updates will be essential as SARS-CoV-2 continues evolving in immunologically experienced populations.

This study has several limitations. The sample size, while adequate for detecting major differences, may limit detection of subtle variations between subgroups. The pseudotype system used for BA.3.2 testing may not fully recapitulate live virus neutralization. Additionally, the durability of these responses beyond the measured time point remains unknown. Furthermore, pre-vaccination sera were not tested, limiting assessment of fold-change increases following KP.2 vaccination. Here, only serological responses were evaluated; cellular immunity, which may significantly contribute to protection, was not assessed. Despite these limitations, the observed trends in immune escape and antibody quality remain relevant for informing ongoing vaccine updates and public health strategies.

## SUPPLEMENTAL MATERIAL

Supplemental Material mbio.02901-25-s0001.pdf Supplemental methods and Fig. S1.

Table S1 mbio.02901-25-s0002.docx Demographics, SARS-CoV-2 infection, and vaccination summary table of post-KP.2 boosted cohort.

ASM does not own the copyrights to Supplemental Material that may be linked to, or accessed through, an article. The authors have granted ASM a non-exclusive, world-wide license to publish the Supplemental Material files. Please contact the corresponding author directly for reuse.

## References

[1] Link-Gelles R, Chickery S, Webber A, Ong TC, Rowley EAK, DeSilva MB, Dascomb K, Irving SA, Klein NP, Grannis SJ, et al.. 2025. Interim estimates of 2024-2025 COVID-19 vaccine effectiveness among adults aged ≥18 years - VISION and IVY networks, September 2024-January 2025. MMWR Morb Mortal Wkly Rep 74:73–82. doi:10.15585/mmwr.mm7406a1 40014628 PMC11867580

[2] Zhang L, Kempf A, Nehlmeier I, Chen N, Stankov MV, Happle C, Dopfer-Jablonka A, Behrens GMN, Schulz SR, Jäck H-M, Pöhlmann S, Hoffmann M. 2025. Host cell entry and neutralisation sensitivity of SARS-CoV-2 BA.3.2. Lancet Microbe:101165. doi:10.1016/j.lanmic.2025.101165 40480242

[3] Guo C, Yu Y, Liu J, Jian F, Yang S, Song W, Yu L, Shao F, Cao Y. 2025. Antigenic and virological characteristics of SARS-CoV-2 variants BA.3.2, XFG, and NB.1.8.1. Lancet Infect Dis 25:e374–e377. doi:10.1016/S1473-3099(25)00308-1 40484018

[4] Wilks SH, Mühlemann B, Shen X, Türeli S, LeGresley EB, Netzl A, Caniza MA, Chacaltana-Huarcaya JN, Corman VM, Daniell X, et al.. 2023. Mapping SARS-CoV-2 antigenic relationships and serological responses. Science 382:eadj0070. doi:10.1126/science.adj0070 37797027 PMC12145880

[5] Smith DJ, Lapedes AS, de Jong JC, Bestebroer TM, Rimmelzwaan GF, Osterhaus ADME, Fouchier RAM. 2004. Mapping the antigenic and genetic evolution of influenza virus. Science 305:371–376. doi:10.1126/science.1097211 15218094

[6] Keeton R, Tincho MB, Ngomti A, Baguma R, Benede N, Suzuki A, Khan K, Cele S, Bernstein M, Karim F, et al.. 2022. T cell responses to SARS-CoV-2 spike cross-recognize Omicron. Nature 603:488–492. doi:10.1038/s41586-022-04460-3 35102311 PMC8930768

[7] Tarke A, Coelho CH, Zhang Z, Dan JM, Yu ED, Methot N, Bloom NI, Goodwin B, Phillips E, Mallal S, Sidney J, Filaci G, Weiskopf D, da Silva Antunes R, Crotty S, Grifoni A, Sette A. 2022. SARS-CoV-2 vaccination induces immunological T cell memory able to cross-recognize variants from Alpha to Omicron. Cell 185:847–859. doi:10.1016/j.cell.2022.01.015 35139340 PMC8784649

[8] Slamanig S, González-Domínguez I, Chang LA, Lemus N, Lai TY, Martínez JL, Singh G, Dolange V, Abdeljawad A, Kowdle S, Noureddine M, Warang P, Singh G, Lee B, García-Sastre A, Krammer F, Schotsaert M, Palese P, Sun W. 2024. Intranasal SARS-CoV-2 Omicron variant vaccines elicit humoral and cellular mucosal immunity in female mice. EBioMedicine 105:105185. doi:10.1016/j.ebiom.2024.105185 38848648 PMC11200293

