# Supplementary material for: Antibody responses to SARS-CoV-2 variants LP.8.1, LF.7.1, NB.1.8.1, XFG, and BA.3.2 following KP.2 monovalent mRNA vaccination
Source: mBio. 2025 Nov 25;17(1):e02901-25. doi: 10.1128/mbio.02901-25 (PMC12802213; doi:10.1128/mbio.02901-25)
Supplement: Supplemental Material — Supplemental methods and Fig. S1. [file mbio.02901-25-s0001.pdf]

Methods

Human cohort description

De-identified human serum samples were collected from 56 healthy participants enrolled in the following observational studies: PARIS (Protection Associated with Rapid Immunity to SARS-CoV-2, IRB20-03374) and the observational longitudinal clinical sample collection from patients with emerging viral infections (IRB-17-00791/STUDY-16-01215) at the Icahn School of Medicine at Mount Sinai. All participants received KP.2 monovalent mRNA vaccines and were stratified based on their SARS-CoV-2 exposure history. These studies were approved by the Mount Sinai Hospital Institutional Review Board and all participants provided written informed consent prior to sample and data collection. Information regarding COVID-19 vaccination, SARS-CoV-2 infection were participant reported. Demographics and immune history analysis are shown in Table 1.

Cell culture

Vero-E6 cells expressing transmembrane protease serine 2 (TMPRSS2) (BPS Biosciences, cat. no. 78081) were maintained in Dulbecco's modified Eagle's medium (DMEM; Gibco, cat. no. 11965092) supplemented with 10% heat-inactivated fetal bovine serum (FBS; Gibco, cat. no. A5256801), 1% minimum essential medium with non-essential amino acids (Gibco, cat. no. 11140050), 100 U/mL penicillin and 100 µg/mL streptomycin (Gibco, cat. no. 15140122), 100 µg/mL normocin (InvivoGen, cat. no. ant-nr), and 3 µg/mL puromycin (InvivoGen, cat. no. ant-pr). Baby hamster kidney cells expressing angiotensin converting enzyme 2 (BHK-ACE2) cells were cultured in DMEM supplemented with 10% FBS and 1% penicillin/streptomycin. Expi293F cells (Gibco, cat. no. A14527) were maintained in Expi293 Expression Medium (Gibco, cat. no. A1435102).

Replication competent SARS-CoV-2 isolates

The SARS-CoV-2 isolate USA-WA.1/2020 was used as a wild-type/ancestral reference (BEI Resources; NR-52281). The following viral isolates from the Omicron lineage were provided by the Mount Sinai Pathogen Surveillance Program: hCoV-19/USA/NY-MSHSPSP-PV96109/2023 (JN.1), hCoV-19/USA/NY-MSHSPSP-PV112116/2024 (KP.2), hCoV-19/USA/NY-MSHSPSP-PV301922/2024 (LP.8.1), hCoV-19/USA/NY-MSHSPSP-PV301501/2025 (LF.7.1), hCoV-19/USA/NY-MSHSPSP-PV304487/2025 (NB.1.8.1), and hCoV-19/USA/NY-MSHSPSP-PV304164/2025 (XFG).

Replication competent SARS-CoV-2 neutralization assay

Neutralizing antibody titers against SARS-CoV-2 variants (WA.1, JN.1, KP.2, LP.8.1, LF.7.1, NB.1.8.1, and XFG) were measured using a multicycle microneutralization assay in a BSL-3 facility. Vero-E6 TMPRSS2 cells (2×10^5 cells/well) were seeded in 96-well plates 24 hours prior to infection. Heat-inactivated sera were 3-fold serially diluted starting at 1:10 in modified Eagle's medium (1xMEM) and incubated with 10,000 50% tissue culture infectious dose ($TCID_{50}$) of each

virus for 1 hour at room temperature. Virus-serum mixtures were transferred to cell plates and incubated for 1 hour at 37°C. After removing inoculum, 1xMEM supplemented with 2% FBS was added, and plates were incubated for 48 hours at 37°C. Cells were fixed with 10% formaldehyde overnight at 4°C, permeabilized with 0.1% Triton X-100, and blocked with 3% bovine serum albumin (BSA) in PBS. Biotinylated anti-SARS-CoV nucleoprotein mAb 1C7C7 (1 μg/mL) was added for 2 hour, followed by HRP-conjugated streptavidin (1:2,000) for 1 hour. OPD substrate was added for 10 minutes, stopped with 3 M HCl, and optical density was measured at 490 nm. The 50% inhibitory dilution (ID_{50}) was calculated using non-linear regression analysis with 100% and 0% constraints.

Pseudotyped virus neutralization assay

Neutralizing antibody titers against WA.1 and BA.3.2 variants were measured using replication-competent vesicular stomatitis virus expressing SARS-CoV-2 spike in place of VSV-G (rcVSV-eGFP-CoV2-S) as previously described [2]. Virus stocks were expended on BHK-ACE2 cells and titrated on Vero-E6 TMPRSS2 cells prior to use. One day before the assay, Vero-E6 TMPRSS2 cells (2×10^5 cells/well) were seeded in 96-well plates. Heat-inactivated sera were initially diluted 1:10 in 1× minimum essential medium (MEM) followed by 3-fold serial dilutions. Diluted sera were incubated with 10,000 $TCID_{50}$ of each VSV-spike pseudotyped virus under conditions identical to those used for live virus microneutralization assays. Following 48-hour incubation, cells were fixed and permeabilized as described above. Fixed cell plates were immunostained with anti-VSV-N monoclonal antibody (10G4, cat. no. EB0009) at a final dilution of 1:3,000 for two hours at room temperature, followed by HRP-conjugated anti-mouse secondary antibody (Rockland, cat. no. 610-1102) at a final dilution of 1:3,000 for 1 hour. OPD substrate was added for 10 minutes, the reaction was stopped with 3 M HCl, and optical density was measured at 490 nm. The ID_{50} titers were calculated as described above.

Antigenic cartography

Antigenic maps were constructed from neutralization data using established multidimensional scaling methods as previously described [3,4]. Neutralizing antibody titers were converted to antigenic distances, where each unit corresponds to a 2-fold difference in neutralization capacity. Variants and sera were positioned in multidimensional space such that Euclidean distances between points accurately reflect antigenic relationships. Maps were optimized to minimize stress between observed and predicted neutralization titers, then projected into two dimensions for visualization. Antigenic cartography maps were generated in R (version 4.3.3) using the Racmacs package (version 1.2.9) [3]. The number of optimization runs was set to 3,000, and the minimum column basis was set to none to allow for maximum flexibility in map construction. The limit of detection (LoD) for neutralizing antibody titers was established at 1:10, and all titers below this threshold were assigned as "<10" in the titer table for subsequent analysis.

Enzyme-linked immunosorbent assay (ELISA)

IgG binding antibody titers against SARS-CoV-2 NP were measured using an in-house ELISA. Briefly, 96-well microtiter plates (Immulon 4 HBX; Thermo Scientific, cat. no. 439454) were coated overnight at 4°C with 2 μg/mL recombinant spike protein. After washing with 1× phosphate

buffered saline supplement with 0.1 % Tween 20 (PBS-T) and blocking with 3% milk powder in PBS-T for 1 hour at room temperature, heat-inactivated sera were added in 2-fold serial dilutions starting at 1:100. Following 2-hour incubation and washing, anti-human IgG (Fab-specific) horseradish peroxidase (HRP)-conjugated secondary antibody (Sigma-Aldrich, cat. no. A0293) was added at 1:3,000 dilution. After 1-hour incubation and washing, o-phenylenediamine dihydrochloride substrate (SIGMAFAST) was added for 10 minutes, and the reaction was stopped with 3 M HCl. Optical density was measured at 490 nm using a Synergy H1 microplate reader (BioTek), and area under the curve (AUC) values were calculated using Prism 10 (GraphPad).

Statistical analysis

Statistical analyses were performed using Prism 10 software (GraphPad). Log-transformed neutralization titers were compared between groups. Normality was assessed using Shapiro-Wilk tests. For non-normally distributed data, Kruskal-Wallis test with Dunn's multiple comparisons test was applied. Wilcoxon matched-pairs signed rank test was used for paired comparisons. Geometric mean titers and geometric mean ratios with 95% confidence intervals were calculated for all analyses. Statistical significance was defined as $p < 0.05$.

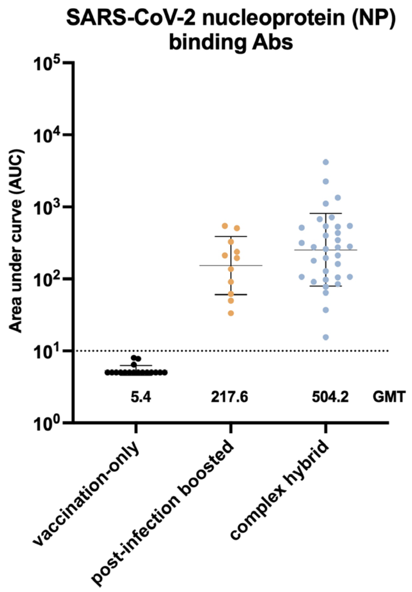

Fig. S1. NP binding antibody responses for the overall cohort by exposure group.
