# Supplementary material for: Antibody responses to SARS-CoV-2 variants LP.8.1, LF.7.1, NB.1.8.1, XFG, and BA.3.2 following KP.2 monovalent mRNA vaccination
Source: mBio. 2025 Nov 25;17(1):e02901-25. doi: 10.1128/mbio.02901-25 (PMC12802213; doi:10.1128/mbio.02901-25)
Supplement: Table S1 — Demographics, SARS-CoV-2 infection, and vaccination summary table of post-KP.2 boosted cohort. [file mbio.02901-25-s0002.docx]

**Table S1. Demographics, SARS-CoV-2 infection and vaccination summary table of post-KP.2 boosted cohort.** Summary information for a total of 56 individuals collected serum samples (average of 29 days) following mRNA monovalent KP.2 vaccine booster.

|  | **Vaccination Only** | **Post-Infection Boosted** | **Complex Hybrid Immunity** | **Total** |
| --- | --- | --- | --- | --- |
| **N total** | 16 | 11 | 29 | 56 |
| **Age** | avg (min – max) | avg (min – max) | avg (min – max) | avg (min – max) |
|  | 44.8 (20 – 75) | 40.5 (25 – 62) | 41.4 (23 – 81) | 42.2 (20 – 81) |
| **Sex at Birth** | n (% group) | n (% group) | n (% group) | n (% total) |
| **Female** | 11 (68.75%) | 8 (72.73%) | 23 (79.31%) | 42 (75%) |
| **Male** | 5 (31.25%) | 3 (27.27%) | 6 (20.69%) | 14 (25%) |
| **Bivalent Boost** | n (% group) | n (% group) | n (% group) | n (% total) |
| **Yes** | 11 (68.75%) | 10 (90.91%) | 19 (65.52%) | 40 (71.43%) |
| **No** | 5 (31.25%) | 1 (9.09%) | 10 (34.48%) | 16 (28.57%) |
| **XBB Boost** | n (% group) | n (% group) | n (% group) | n (% total) |
| **Yes** | 9 (56.25%) | 6 (54.55%) | 24 (82.76%) | 39 (69.64%) |
| **No** | 7 (43.75%) | 5 (45.45%) | 5 (17.24%) | 17 (30.36%) |
| **Infection** | n (% group) | n (% group) | n (% group) | n (% total) |
| **Yes** | 0 (0%) | 11 (100%) | 29 (100%) | 42 (75%) |
| **No** | 16 (100%) | 0 (0%) | 0 (0%) | 14 (25%) |
| **KP.2 vaccination type** | n (% group) | n (% group) | n (% group) | n (% total) |
| **Pfizer** | 12 (75%) | 4 (36.36%) | 19 (65.52%) | 35 (62.5%) |
| **Moderna** | 4 (25%) | 7 (63.64%) | 10 (34.48%) | 21 (37.5%) |
